# Adjuvant nivolumab versus placebo following radical surgery for high-risk muscle-invasive urothelial carcinoma: a subgroup analysis of Japanese patients enrolled in the phase 3 CheckMate 274 trial

**DOI:** 10.1093/jjco/hyac155

**Published:** 2022-10-26

**Authors:** Yoshihiko Tomita, Ko Kobayashi, Go Kimura, Mototsugu Oya, Hirotsugu Uemura, Hiroyuki Nishiyama, Matthew D Galsky, Federico Nasroulah, Sandra Collette, Edward Broughton, Keziban Ünsal-Kaçmaz, Yukinori Kamisuki, Dean F Bajorin

**Affiliations:** Department of Urology, Molecular Oncology, Niigata University Graduate School of Medical and Dental Sciences, Niigata, Japan; Department of Urology, School of Medicine, Sapporo Medical University, Sapporo, Japan; Department of Urology, Nippon Medical School Hospital, Tokyo, Japan; Department of Urology, Keio University School of Medicine, Tokyo, Japan; Department of Urology, Kindai University Hospital, Faculty of Medicine, Osaka, Japan; Department of Urology, Faculty of Medicine, University of Tsukuba, Ibaraki, Japan; Icahn School of Medicine at Mount Sinai, New York, NY, USA; Bristol-Myers Squibb, Princeton, NJ, USA; Bristol-Myers Squibb, Princeton, NJ, USA; Bristol-Myers Squibb, Princeton, NJ, USA; Bristol-Myers Squibb, Princeton, NJ, USA; Ono Pharmaceutical Co., Ltd., Osaka, Japan; Memorial Sloan Kettering Cancer Center, New York, NY, USA

**Keywords:** nivolumab, adjuvant chemotherapy, urinary bladder neoplasms, carcinoma, Japanese

## Abstract

**Background:**

The phase 3 CheckMate 274 trial demonstrated superiority of adjuvant nivolumab over placebo after radical surgery in patients with high-risk muscle-invasive urothelial carcinoma. However, the efficacy and safety of adjuvant nivolumab in Japanese patients with muscle-invasive urothelial carcinoma have not been clarified.

**Methods:**

Patients with muscle-invasive urothelial carcinoma were randomized to adjuvant nivolumab 240 mg or placebo (every 2 weeks via intravenous infusion) up to 120 days after radical surgery in CheckMate 274.

**Results:**

Of 49 patients in the Japanese subgroup, 27 and 22 patients were randomized to nivolumab and placebo, respectively. Eleven and 8 patients, respectively, had tumor PD-L1 expression level of 1% or more. The median disease-free survival times in the nivolumab and placebo groups were 29.67 months (95% confidence interval 7.79–not reached) and 9.72 months (95% confidence interval 4.73–not reached), respectively (hazard ratio 0.77, 95% confidence interval 0.35–1.69). The corresponding values in patients with tumor PD-L1 expression level of 1% or more were 29.67 months (95% confidence interval 2.63–not reached) and 25.95 months (95% confidence interval 5.59–not reached) (hazard ratio 1.10, 95% confidence interval 0.31–3.92), respectively. Treatment-related adverse events of Grade 3–4 occurred in 25.9 and 13.6% of patients in the nivolumab and placebo groups, respectively. The most common treatment-related adverse events in the nivolumab group were lipase increased, amylase increased and diarrhea. The changes in quality of life scores from baseline over time were similar in both groups.

**Conclusions:**

The efficacy and safety results in the Japanese subgroup were consistent with the overall population of CheckMate 274.

## Teaser

Adjuvant nivolumab improved disease-free survival in Japanese patients, with no impairment in quality of life and a safety profile consistent with that of the overall study population of CheckMate 274.

## Introduction

The standard treatment for muscle-invasive urothelial carcinoma (MIUC) is radical surgery (1,2). The type of surgery depends on the location of the carcinoma: cystectomy for bladder carcinoma and nephroureterectomy for upper tract urothelial carcinomas (UTUC) (3,4). Despite radical surgery, it has been estimated that ~50% of patients experience metastatic recurrence within 1 year, and 5-year survival rates of patients with pT3 or pT4 of 31–38 and 21–33%, respectively, have been reported (5,6).

Neoadjuvant cisplatin-based chemotherapy is now established as a standard treatment in the clinical practice guidelines for bladder cancer (1–3), and its survival benefit has been demonstrated in several meta-analyses (7,8). Kitamura *et al.* (9) also demonstrated its usefulness in Japanese patients. However, the outcomes of neoadjuvant chemotherapy are unsatisfactory, with 5-year survival rates that ranged from 20 to 40% in patients with urothelial carcinomas (UC) graded pT2 or worse or patients with lymph node metastasis (N+) (10). Although the timing of adjuvant chemotherapy (immediate/deferred) has been trialed in patients with pT3–4 or N+ M0 UC of the bladder, the results remain suboptimal; although immediate chemotherapy (within 90 days after surgery) was associated with improved progression-free survival (PFS) compared with deferred chemotherapy until first recurrence (5-year PFS: 47.6 vs. 31.8%), overall survival (OS) was not significantly improved with immediate chemotherapy (11). A large retrospective study reported that the 5-year OS rate was significantly better in patients who received adjuvant chemotherapy compared with patients who underwent observation following radical cystectomy without neoadjuvant chemotherapy (37.0 vs. 29.1%, *P* < 0.001) (12). An updated analysis further confirmed a benefit of cisplatin-based adjuvant chemotherapy on OS (56.0 vs. 50.0%) (13). However, compared with neoadjuvant chemotherapy, there is limited evidence supporting adjuvant chemotherapy for bladder cancer and it is not considered standard therapy in Japan (3). Adjuvant chemotherapy may prolong disease-free survival (DFS) in patients with locally advanced UTUC eligible for chemotherapy (14). However, decreased renal function has been reported after nephroureterectomy (15) and may contraindicate chemotherapy. Therefore, the Japanese guidelines state that when cisplatin-based chemotherapy is used for UTUC, it should be performed as neoadjuvant therapy, but no specific chemotherapy regimen has been proposed (4). For these reasons, there remains a need for a novel and effective perioperative therapy for MIUC.

Immune checkpoint inhibitors that target programmed death 1 or programmed death ligand 1 (PD-L1) have shown clinical activity in patients with advanced UC (1,16–19). Avelumab maintenance therapy is recommended as the standard of care after first-line anticancer chemotherapy for patients with unresectable or metastatic disease, and pembrolizumab is recommended as the standard of care for patients with locally advanced or metastatic urothelial carcinoma who have disease progression during or following platinum-containing chemotherapy or within 12 months of neoadjuvant or adjuvant treatment with platinum-containing chemotherapy (1–3). However, there are no reports describing immune checkpoint inhibitors in the perioperative period in Japan.

The phase 3 CheckMate 274 trial was performed to verify the superiority of adjuvant nivolumab over placebo in patients with high-risk MIUC after radical cystectomy (20). Nivolumab reduced the risk of recurrence or death by 30% compared with placebo (hazard ratio [HR] 0.70; 98.22% confidence interval [CI] 0.55–0.90). This benefit was also observed in a subset of patients with tumors positive for PD-L1 in whom nivolumab reduced the risk of recurrence or death by 45% compared with placebo (HR 0.55; 98.72% CI 0.35–0.85). CheckMate 274 identified no additional safety signals beyond the previous studies of nivolumab monotherapy. Additionally, according to the changes from baseline in the European Organization for Research and Treatment of Cancer Quality of Life Questionnaire-Core 30 (EORTC QLQ-C30) Global Health Status score, there was no meaningful difference in the deterioration in quality of life (QOL) between patients who received nivolumab and those who received placebo in the intention-to-treat (ITT) population and in patients with a PD-L1 expression level of 1% or more.

Because CheckMate 274 enrolled 49 Japanese patients (27 patients in the nivolumab group and 22 patients in the placebo group), we performed a subgroup analysis of these patients to verify the efficacy and safety of nivolumab in Japanese patients. This is the first report to describe the efficacy and tolerability outcomes of nivolumab as adjuvant therapy in Japanese patients with MIUC.

## Methods

The design of CheckMate 274 has been described previously (20). The trial was approved by the institutional review boards at each participating site and complied with ethical guidelines, including Good Clinical Practice (GCP), International Conference on Harmonization-GCP and the Declaration of Helsinki. The trial was registered on ClinicalTrials.gov (accession number: NCT02632409).

### Patients and treatments

Patients were eligible for the trial if they had undergone radical surgery (R0 with negative surgical margins) up to 120 days before randomization for pathologically confirmed MIUC arising in the bladder, ureter or renal pelvis and if they were considered at high risk of recurrence [pathological stage of pT3, pT4a or pN+ and patient not eligible for (21) or declined adjuvant cisplatin-based combination chemotherapy for patients who had not received neoadjuvant cisplatin-based chemotherapy, and pathological stage of ypT2 to ypT4a or ypN+ for patients who received neoadjuvant cisplatin]. Patients with or without cisplatin-based neoadjuvant chemotherapy were eligible. All patients must have been disease-free within 4 weeks of randomization and have an Eastern Cooperative Oncology Group (ECOG) performance status (PS) of 0 or 1. The eligibility criteria are described in further detail in the previous report (20). In analyses of CheckMate 274, Japanese patients were included under the classification ‘Asians.’

Eligible patients were randomized to either nivolumab or placebo (1:1 ratio) with stratification by tumor PD-L1 expression (1% or more vs. less than 1%/indeterminate), pathological nodal status (N+ vs. N0 or NX with fewer than 10 nodes removed vs. N0 with 10 or more nodes removed) and cisplatin-based neoadjuvant chemotherapy (yes vs. no). Japanese was not included as a stratification factor. PD-L1 expression was determined using the PD-L1 IHC 28-8 pharmDx immunohistochemical assay (Dako).

Patients were treated with 240 mg nivolumab or placebo every 2 weeks via intravenous infusion (30 min duration). Treatment was continued for up to 1 year or until discontinuation for disease recurrence. Dose delays or discontinuations were permitted if deemed necessary to manage adverse events (AEs).

### Outcomes

The two primary endpoints were DFS among all randomized patients (ITT population) and DFS among patients with a tumor PD-L1 expression level of 1% or more. DFS was defined as the time from randomization to the date of first recurrence (local recurrence within the urothelial tract, local recurrence outside the urothelial tract or distant recurrence) or death (from any cause), whichever occurred first. Recurrence was assessed by the investigators. The study protocol encouraged biopsy if feasible and deemed appropriate by the investigator.

Secondary endpoints included non-urothelial tract recurrence-free survival (NUTRFS), OS and disease-specific survival in both trial populations. NUTRFS was defined as the time from randomization to the date of first documented recurrence (local non-urothelial tract or distant) or death (from any cause), whichever occurred first.

Exploratory endpoints included safety, side effects, distant metastasis-free survival and QOL. QOL was assessed using the EORTC QLQ-C30 (22). AEs were evaluated and graded according to the Common Terminology Criteria for Adverse Events version 4.0 (23). The relationship between AEs and the study drug was determined by the investigators.

### Statistical analyses

Efficacy data were analyzed using all randomized patients and randomized patients with tumor PD-L1 expression level of 1% or more, and safety data were analyzed using all patients who received the study drug at least once.

DFS was analyzed using the unstratified log-rank test and an overall type I error of 2.5% (two-sided) for between-group comparisons in each patient population (all randomized patients and patients with tumor PD-L1 expression of 1% or more). The HRs and corresponding CIs for DFS and NUTRFS were estimated using the unstratified Cox proportional hazard model. DFS curves were plotted using the Kaplan–Meier (KM) method, and the median DFS with 95% CI (two-sided) was calculated using double-logarithmic conversion.

The EORTC QLQ-C30 scores all scales and single items on a categorical scale with a linear transformation to a 0–100 scale. Higher scores on the Global Health Status scale represent higher levels of global health status. For the EORTC QLQ-C30 scale, a score difference of 10 is considered to be a clinically important difference (24). The mean scores and mean changes from baseline for the EORTC QLQ-C30 Global Health Status were summarized using descriptive statistics for all scales at each time point. Line graphs summarizing the mean change from baseline were plotted for each scale.

CheckMate 274 was not prospectively designed to detect differences between the treatment groups among Japanese patients.

## Results

### Patients

Of 709 patients randomized in CheckMate 274 (353 to nivolumab and 356 to placebo), 49 were Japanese and included in the present analyses. Twenty-seven were allocated to nivolumab and 22 to placebo per the central randomization process.

The characteristics of the Japanese patients and the overall population are summarized in Tables 1 and 2, respectively. Some differences between the nivolumab and placebo groups were apparent because the stratification factors did not include Japanese. In particular, the nivolumab group included higher percentages of patients with an ECOG PS of 1 or more (nivolumab vs. placebo: 14.8 vs. 0%), tumor originating in the bladder (48.1 vs. 22.7%) and N+ node status (51.9 vs. 27.3%). The main differences in patient characteristics between the overall and Japanese populations were as follows: ECOG PS of 0 (63.5 and 85.2%, respectively) and tumor originating in the bladder (79.0 and 48.1%, respectively).

**Table 1:** Demographic and clinical characteristics of all randomized Japanese patients at baseline^a^

Characteristic	Nivolumab (*n* = 27)	Placebo (*n* = 22)
Age		
Mean (range)—years	68.8 (49–92)	67.3 (45–76)
<65 years—no. (%)	10 (37.0)	5 (22.7)
≥65 years—no. (%)	17 (63.0)	17 (77.3)
Sex—no. (%)		
Male	18 (66.7)	15 (68.2)
Female	9 (33.3)	7 (31.8)
Race or ethnic group—no. (%)^b^		
Asian	27 (100.0)	22 (100.0)
ECOG PS—no. (%)^c^		
0	23 (85.2)	22 (100.0)
1	4 (14.8)	0
2	0	0
Tumor origin at initial diagnosis—no. (%)		
Urinary bladder	13 (48.1)	5 (22.7)
Renal pelvis	10 (37.0)	14 (63.6)
Ureter	4 (14.8)	3 (13.6)
Time from initial diagnosis to randomization—no. (%)		
<1 year	26 (96.3)	21 (95.5)
≥1 year	1 (3.7)	1 (4.5)
Minor histological variants present—no. (%)	5 (18.5)	4 (18.2)
PD-L1 expression of ≥1% (IVRS)—no. (%)	11 (40.7)	8 (36.4)
Prior neoadjuvant cisplatin—no. (%)	12 (44.4)	11 (50.0)
Baseline creatinine clearance—no. (%)		
<60 ml/min	17 (63.0)	17 (77.3)
≥60 ml/min	10 (37.0)	5 (22.7)
Not reported	0	0
Smoking status—no. (%)		
Current/former	13 (48.1)	15 (68.2)
Never	14 (51.9)	7 (31.8)
Unknown	0	0
Pathological tumor stage at resection—no. (%)^d^		
pTX	2 (7.4)	0
pT0	0	0
pTis	1 (3.7)	0
pT1	1 (3.7)	0
pT2	1 (3.7)	0
pT3	19 (70.4)	19 (86.4)
pT4a	3 (11.1)	3 (13.6)
Not reported	0	0
Nodal status at resection—no. (%)^e^		
N0 or NX with <10 nodes removed	10 (37.0)	11 (50.0)
N0 with ≥10 nodes removed	3 (11.1)	5 (22.7)
N1	9 (33.3)	2 (9.1)
N2	4 (14.8)	4 (18.2)
N3	1 (3.7)	0
Not reported	0	0

ECOG PS, Eastern Cooperative Oncology Group performance status; PD-L1, programmed death ligand 1; IVRS, interactive voice-response system.

^a^Percentages may not total 100 because of rounding.

^b^Race or ethnic group was reported by the patient.

^c^ECOG PS scores range from 0 to 5, with higher scores indicating greater disability.

^d^This was not a prespecified subgroup. Patients with pT2N−disease were eligible only if they received neoadjuvant cisplatin-based chemotherapy. N− includes N0 and NX, and T0 includes pTX, pT0 and pTis.

^e^The pathological tumor staging included patients with any nodal status.

**Table 2:** Demographic and clinical characteristics of all randomized patients at baseline^a^

Characteristic	Nivolumab (*n* = 353)	Placebo (*n* = 356)
Age		
Mean (range)—years	65.3 (30–92)	65.9 (42–88)
<65 years—no. (%)	155 (43.9)	136 (38.2)
≥65 years—no. (%)	198 (56.1)	220 (61.8)
Sex—no. (%)		
Male	265 (75.1)	275 (77.2)
Female	88 (24.9)	81 (22.8)
Race or ethnic group—no. (%)^b^		
White	264 (74.8)	272 (76.4)
Asian	80 (22.7)	75 (21.1)
Black	2 (0.6)	3 (0.8)
American Indian or Alaska Native	1 (0.3)	0
Other	6 (1.7)	5 (1.4)
Not reported	0	1 (0.3)
ECOG performance-status score—no. (%)^c^		
0	224 (63.5)	221 (62.1)
1	122 (34.6)	125 (35.1)
2	7 (2.0)	9 (2.5)
Not reported	0	1 (0.3)
Tumor origin at initial diagnosis—no. (%)		
Urinary bladder	279 (79.0)	281 (78.9)
Renal pelvis	44 (12.5)	52 (14.6)
Ureter	30 (8.5)	23 (6.5)
Time from initial diagnosis to randomization—no. (%)		
<1 year	325 (92.1)	324 (91.0)
≥1 year	28 (7.9)	32 (9.0)
PD-L1 expression of ≥1% (IVRS)—no. (%)	140 (39.7)	142 (39.9)
Previous neoadjuvant cisplatin therapy—no. (%)	153 (43.3)	155 (43.5)
Pathological tumor stage and nodal status at resection—no. (%)^d^		
pT2N–	25 (7.1)	29 (8.1)
pT3,4N–	158 (44.8)	159 (44.7)
pT0–4N1	71 (20.1)	72 (20.2)
pT0–4N2,3	96 (27.2)	96 (27.0)
pTisN–	1 (0.3)	0
Not reported	2 (0.6)	0
Pathological tumor stage at resection—no. (%)^e^		
pTX	5 (1.4)	0
pT0	5 (1.4)	7 (2.0)
pTis	4 (1.1)	3 (0.8)
pT1	13 (3.7)	14 (3.9)
pT2	62 (17.6)	65 (18.3)
pT3	206 (58.4)	204 (57.3)
pT4a	57 (16.1)	62 (17.4)
Not reported	1 (0.3)	1 (0.3)
Nodal status at resection		
N0 or NX with <10 nodes removed	94 (26.6)	99 (27.8)
N0 with ≥10 nodes removed	91 (25.8)	88 (24.7)
N1	71 (20.1)	72 (20.2)
N2	84 (23.8)	76 (21.3)
N3	12 (3.4)	20 (5.6)
Not reported	1 (0.3)	1 (0.3)

Reprinted from Ref. (20). Copyright © 2021 Massachusetts Medical Society. All rights reserved.

^a^Percentages may not total 100 because of rounding. IVRS denotes interactive voice-response system, and PD-L1 programmed death ligand 1.

^b^Race or ethnic group was reported by the patient.

^c^Eastern Cooperative Oncology Group (ECOG) performance-status scores range from 0 to 5, with higher scores indicating greater disability.

^d^This was not a prespecified subgroup. Patients with pT2N− disease were eligible only if they received neoadjuvant cisplatin-based chemotherapy. N− includes N0 and NX, and T0 includes pTX, pT0 and pTis.

^e^The pathological tumor staging included patients with any nodal status.

In the Japanese cohort, tumor PD-L1 expression level was 1% or more in 11 patients in the nivolumab group and in 8 patients in the placebo group. Some numerical differences were observed between these two groups for the following baseline characteristics: age 65 years or greater, ECOG PS, cancer location, lymph node status and cisplatin-based neoadjuvant chemotherapy (Supplementary Table S1). Corresponding data in the overall population are shown in Supplementary Table S2.

### Disease-free survival

**Figure 1:**
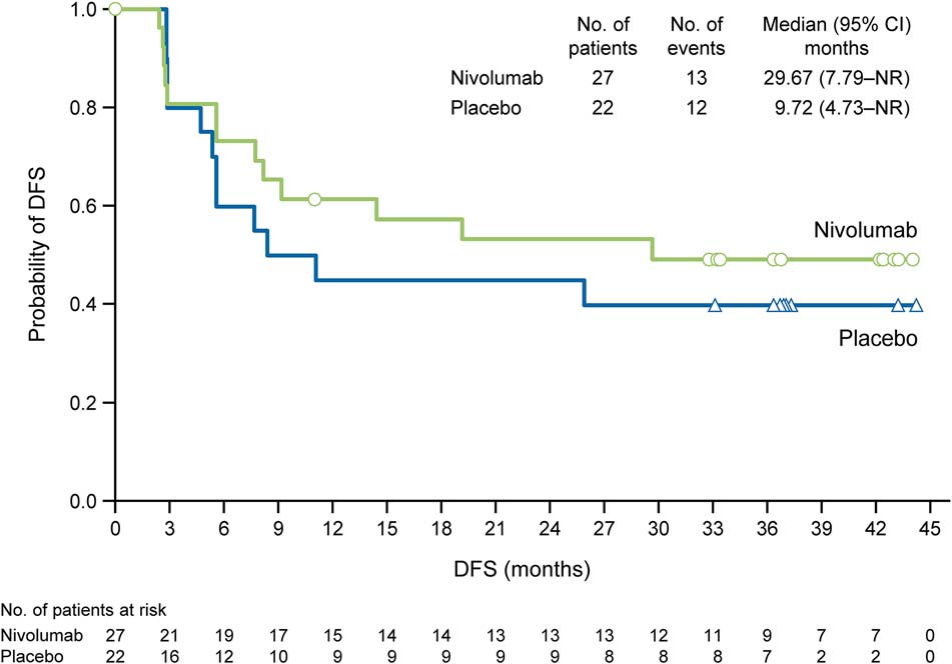
DFS according to treatment in all randomized Japanese patients. CI, confidence interval; DFS, disease-free survival; NR, not reached.

Among Japanese patients, the median DFS was 29.67 months (95% CI 7.79–not reached [NR]) in the nivolumab group and 9.72 months (95% CI 4.73–NR) in the placebo group (Fig. 1). The HR for nivolumab relative to placebo was 0.77 (95% CI 0.35–1.69; Cox proportional hazard model).

**Figure 2:**
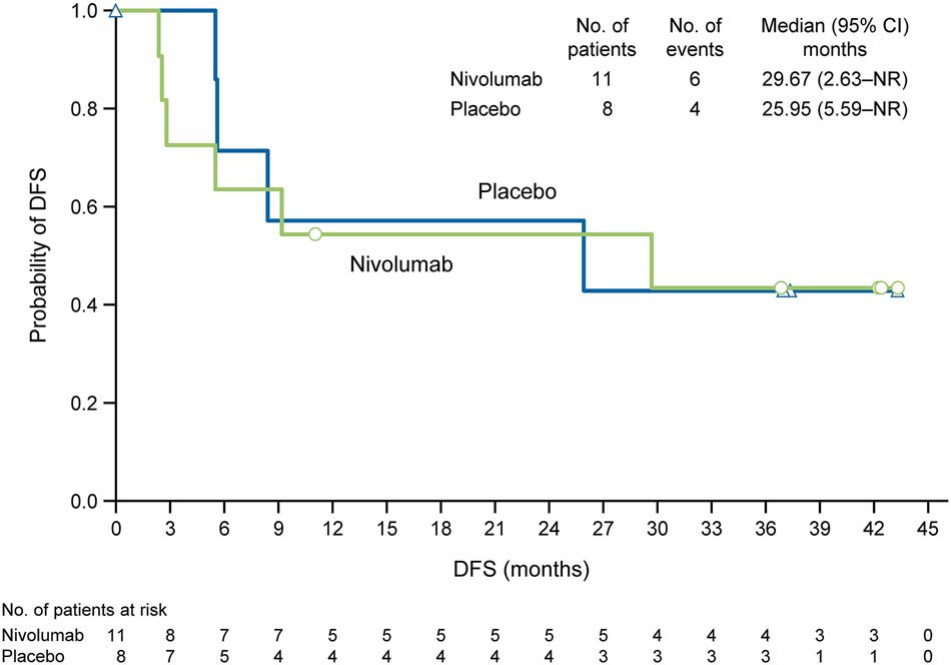
DFS according to treatment in Japanese patients with tumor programmed death ligand 1 expression of 1% or more. CI, confidence interval; DFS, disease-free survival; NR, not reached.

Among patients with tumor PD-L1 expression level of 1% or more (nivolumab, *n* = 11; placebo, *n* = 8), the median DFS was 29.67 months (95% CI 2.63–NR) in the nivolumab group and 25.95 months (95% CI 5.59–NR) in the placebo group (Fig. 2). The HR for nivolumab relative to placebo was 1.10 (95% CI 0.31–3.92; Cox proportional hazard model).

**Figure 3:**
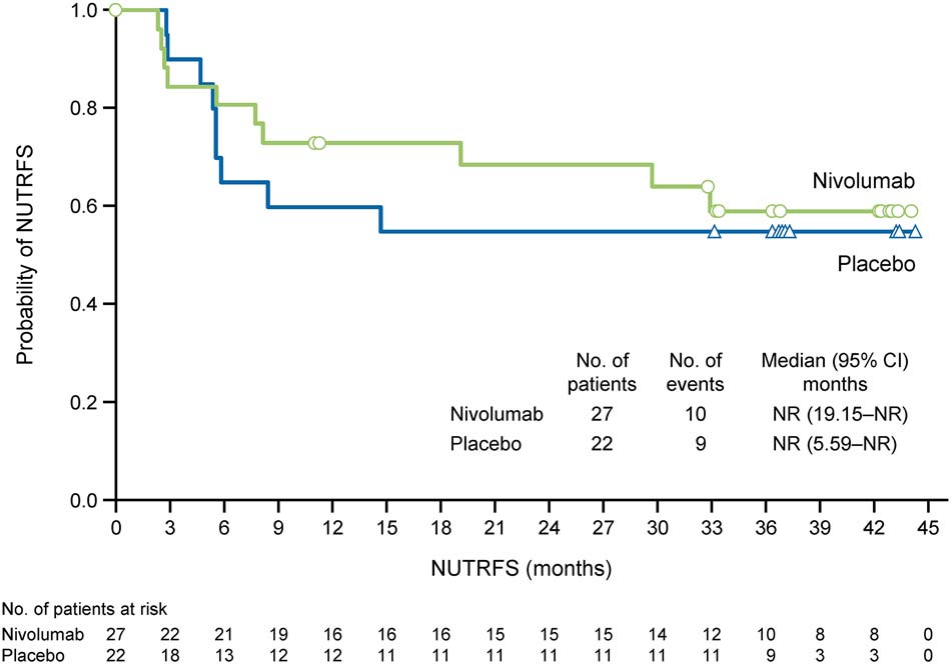
NUTRFS according to treatment in all randomized Japanese patients. CI, confidence interval; NR, not reached; NUTRFS, non-urothelial tract recurrence-free survival.

**Figure 4:**
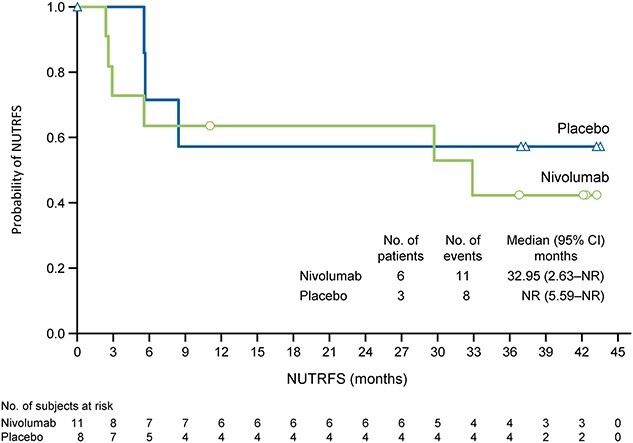
NUTRFS according to treatment in Japanese patients with tumor programmed death ligand 1 expression of 1% or more. CI, confidence interval; NR, not reached; NUTRFS, non-urothelial tract recurrence-free survival.

### NUTRFS

Figure 3 shows the KM curves of NUTRFS in Japanese patients. The median NUTRFS was NR in the nivolumab group (95% CI 19.15–NR) and the placebo group (95% CI 5.59–NR). The HR for nivolumab relative to placebo was 0.84 (95% CI 0.34–2.06; Cox proportional hazard model).

Among Japanese patients with tumor PD-L1 expression level of 1% or more (nivolumab, *n* = 11; placebo, *n* = 8), the median NUTRFS was 32.95 months (95% CI 2.63–NR) in the nivolumab group and not reached (95% CI 5.59−NR) in the placebo group. Figure 4 shows the KM curves. The HR for nivolumab relative to placebo was 1.53 (95% CI 0.38–6.12; Cox proportional hazard model).

### Safety

AEs of any cause occurred in 100 and 90.9% of patients in the nivolumab and placebo groups with Grade 3 or higher AEs in 55.6 and 22.7% of patients, respectively (Table 3) Treatment-related adverse events (TRAEs) occurred in 16/27 patients (59.3%) in the nivolumab group, of which 7 (25.9%) experienced Grade 3–4 AEs. TRAEs of any grade occurred in 7/22 patients (31.8%) in the placebo group, of which 3 (13.6%) experienced Grade 3–4 AEs. The common TRAEs in the nivolumab group were lipase increased, amylase increased and diarrhea.

In three patients, TRAEs (one patient with interstitial lung disease, one patient with diarrhea and one patient with colitis, gastroenteritis and white blood cell decreased) were treated with systemic steroids at doses of over 40 mg. One patient experienced Grade 3 interstitial lung disease more than 30 days after discontinuation of nivolumab and was treated with a systemic steroid at a dose of over 40 mg, which is not included in Table 3. There were no Grade 5 TRAEs or deaths within 30 days after the final dose in either group.

**Table 3:** Summary of adverse events

	Nivolumab (*n* = 27)		Placebo (*n* = 22)	
	Any grade	Grade 3–4	Any grade	Grade 3–4
Any AEs	27 (100.0)	15 (55.6)	20 (90.9)	7 (22.7)
Any TRAEs	16 (59.3)	7 (25.9)	7 (31.8)	3 (13.6)
TRAEs leading to treatment discontinuation	4 (14.8)	3 (11.1)	3 (13.6)	1 (4.5)
Type of AE				
Lipase increased	4 (14.8)	4 (14.8)	1 (4.5)	0
Amylase increased	3 (11.1)	1 (3.7)	1 (4.5)	1 (4.5)
Diarrhea	3 (11.1)	1 (3.7)	0	0
Hyponatremia	2 (7.4)	2 (7.4)	0	0
Interstitial lung disease	2 (7.4)	1 (3.7)	0	0
Pruritus	2 (7.4)	0	1 (4.5)	0
Dermatitis	2 (7.4)	0	1 (4.5)	0
Hypothyroidism	2 (7.4)	0	0	0

Values are *n* (%). The table lists AEs that were reported between the first dose and 30 days after the last dose of nivolumab or placebo and occurred in at least 5% of patients in either group. Three patients who developed pulmonary, gastrointestinal or hematological toxicities were treated with systemic steroids at a dose of more than 40 mg. AE, adverse event; TRAE, treatment-related adverse events.

### Quality of life

**Figure 5:**
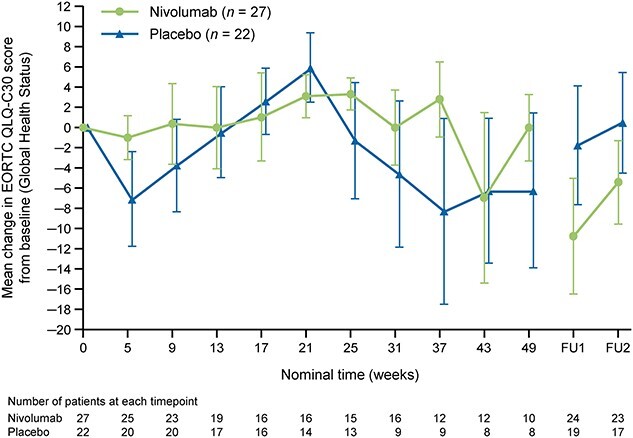
Mean changes in EORTC QLQ-C30 scores for Global Health Status according to treatment in all randomized Japanese patients. EORTC, European Organization for Research and Treatment of Cancer; FU1, follow-up 1; FU2, follow-up 2; QLQ-C30, Quality of Life Questionnaire-Core 30.

QOL was evaluated using the QLQ-C30 questionnaire; the response rate in Japanese patients was 100% in both groups at baseline and at least 92% in each group at each assessment time through to Week 49. As indicated in Fig. 5, both groups were similar and there were no meaningful changes from baseline in the general health QOL scores (defined as a mean change of 10 points or more from baseline) in the Global Health Status in either group at any assessment time. Furthermore, there were no meaningful differences in the deterioration of individual domains (defined as a mean change of 10 points or more from baseline) in either group at nearly all of the assessment times.

## Discussion

This report is the first to describe the efficacy and safety of adjuvant nivolumab in Japanese patients with MIUC enrolled in CheckMate 274, a large phase 3 trial that enrolled 709 patients across 29 countries, including 49 patients from Japan. In all randomized Japanese patients, the median DFS in the nivolumab and placebo groups was 29.67 and 9.72 months, respectively (HR 0.77, 95% CI 0.35–1.69). Despite the bias in patient background, these values are similar to the corresponding values in the ITT population reported previously (20.8 and 10.8 months, respectively; HR 0.70, 98.22% CI 0.55–0.90) (20).

N+ is a risk factor for the recurrence of bladder cancer and UTUC (25–27). The proportion of N+ patients in the nivolumab group in the Japanese population (51.9%) was similar to that in the nivolumab group in the overall population (47.3%). The median DFS in the Japanese population was similar to that in the overall population. In the subgroup analysis of the overall population, the probability of DFS was higher for nivolumab than placebo, regardless of nodal status, and the HR for DFS was 0.64 (95% CI 0.48–0.85) (20). Therefore, similar to the overall population, adjuvant nivolumab is expected to be effective in Japanese patients with N+ MIUC.

Among Japanese patients with tumor PD-L1 expression level of 1% or more, we found no clear difference in DFS between the nivolumab and placebo groups (median 29.67 and 25.95 months, HR 1.10, 95% CI 0.31–3.92) (Fig. 2). By comparison, the DFS rate at 6 months favored nivolumab in the corresponding patients in the overall population with tumor PD-L1 expression level of 1% or more (74.5% with nivolumab and 55.7% with placebo; HR 0.55, 98.72% CI 0.35–0.85) (20). This may be explained by some differences in the characteristics of the placebo group with tumor PD-L1 expression level of 1% or more between the overall population and the Japanese population (including ECOG PS 1 or more, pT3, N+, tumor origin at initial diagnosis) (Supplementary Tables S1 and S2). Regarding patients with tumor PD-L1 expression level of 1% or more, the proportions of patients were lower in the Japanese population than in the overall population for ECOG PS 1 or more (0 and 40.1%, respectively) and N+ (25.0 and 46.5%, respectively), but the proportions of patients were higher for pT3 (87.5 and 58.5%, respectively) and UTUC (75.0 and 17.6%, respectively) (Supplementary Tables S1 and S2). ECOG PS of 1 or more is a prognostic factor for advanced UC (28–30). In addition, lesions graded pT3 or worse and N+ are risk factors for recurrence in patients with bladder cancer and UTUC (31,32). Regarding the relationship between the primary lesion and prognosis, in a previous study of patients with bladder cancer, the 5-year recurrence-free survival (RFS) rates were 62 and 50% in patients with pT3 or pT4 lesions, respectively, at the time of radical resection (6). In patients with UTUC, the 5-year RFS rates were 48 and 4.7% in patients with pT3 or pT4 lesions, respectively, at the time of radical resection (33). This suggests that RFS may be worse in patients with UTUC and lesions graded pT3 or worse than in patients with bladder cancer. However, the impact of these factors on DFS is unknown due to the small sample size of the Japanese subgroup.

A subgroup analysis of the overall population revealed the possibility of a larger effect size in patients with bladder urothelial carcinoma than in those with renal pelvic or ureteral tumors (20). Although UTUC was more frequent in Japanese patients than in the overall population in the nivolumab (51.9 vs. 21.0%) and placebo (77.3 vs. 21.1%) groups, the DFS in the Japanese population was similar to that in the overall population. Therefore, adjuvant nivolumab may be a treatment option for Japanese patients with bladder cancer or UTUC.

CheckMate 274 also documented the safety of nivolumab in patients with MIUC. Of note, the analysis of Japanese patients identified no new safety signals for nivolumab, and the TRAEs were generally controllable by early diagnosis and appropriate therapies. Furthermore, there were no deaths related to the study drug among Japanese patients. In the nivolumab groups, TRAEs occurred in 77.5 and 59.3% of patients in the overall and Japanese populations, respectively, with Grade 3 or higher TRAEs in 17.9 and 25.9% of patients, respectively, and TRAEs leading to treatment discontinuation in 12.8 and 14.8% of patients, respectively (20). These findings suggest that the trends of TRAEs were similar between the overall population and the Japanese population.

Two Japanese patients developed interstitial lung disease (one was considered related to the study drug). Both AEs were controlled and resolved after delaying or discontinuing nivolumab. The incidence of interstitial lung disease in this study was similar to that in prior Japanese studies of nivolumab monotherapy (34–38).

Interstitial lung disease appears to be more common in real-world settings than in clinical trials based on data from post-marketing surveillance of nivolumab-treated patients with non-small cell lung cancer (39). In that analysis, risk factors for interstitial lung disease included previous or comorbid interstitial lung disease, abnormal findings on chest imaging (computed tomography) and a history of smoking. Therefore, urologists and oncologists should be vigilant for interstitial lung disease in patients with MIUC, particularly because smoking is a significant risk factor for bladder cancer.

We should acknowledge that the number of Japanese patients was small, which influenced the numerical frequencies of AEs. Therefore, we should be cautious when comparing the frequencies of AEs between Japanese patients and the overall study population.

No meaningful changes in the EORTC QLQ-C30 scores were observed during treatment in either the Japanese population or the overall population. These results suggest that adjuvant nivolumab is unlikely to affect QOL.

### Limitations

Some limitations of this analysis include the small number of Japanese patients, especially for the analysis of patients with tumor PD-L1 expression of 1% or more. Furthermore, there was an imbalance in the number of patients randomized to nivolumab and placebo in Japan because the stratification factors did not include Japanese. A consequence of this is that some differences in prognostic characteristics between the two groups, including the stratification factors used for randomization, may confound the results.

## Conclusions

This analysis of the Japanese subgroup of patients enrolled in CheckMate 274 was consistent with the overall results of DFS and NUTRFS. We also observed no impairment in QOL, as measured using the EORTC QLQ-C30. The safety profile of nivolumab in Japanese patients was consistent with that in the overall study population, and no new safety concerns were reported. In conclusion, the present results, combined with those of the overall study population, suggest that nivolumab could be useful as adjuvant therapy for Japanese patients with MIUC at high risk of recurrence after radical surgery.

## Conflict of interest

Yoshihiko Tomita reports support for the present manuscript from Ono Pharmaceutical and Bristol-Myers Squibb; consulting fees from Novartis, Ono Pharmaceutical, Taiho Pharmaceutical and MSD; and payment or honoraria from Astellas Pharma, Novartis, Bristol-Myers Squibb and Ono Pharmaceutical.

Ko Kobayashi reports support for the present manuscript from Ono Pharmaceutical and Bristol-Myers Squibb.

Go Kimura reports support for the present manuscript from Ono Pharmaceutical and Bristol-Myers Squibb; consulting fees from Eisai; and payment or honoraria from Ono Pharmaceutical, Merck Biopharma, Chugai, Takeda Pharmaceutical, Bristol-Myers Squibb, MSD and Eisai.

Mototsugu Oya reports support for the present manuscript from Ono Pharmaceutical and Bristol-Myers Squibb and payment or honoraria from Ono Pharmaceutical and Bristol-Myers Squibb.

Hirotsugu Uemura reports support for the present manuscript from Ono Pharmaceutical and Bristol-Myers Squibb; grants from Asahi Kasei Pharma, Janssen Pharmaceutical, Ono Pharmaceutical, Kissei Pharmaceutical, Takeda Pharmaceutical, Sanofi, Daiichi Sankyo, Astellas Pharma and AstraZeneca; consulting fees from Ono Pharmaceutical, Janssen Pharmaceutical and Bayer; and payment or honoraria from Pfizer, Bristol-Myers Squibb, Janssen Pharmaceutical, Merck Biopharma, Bayer, Ono Pharmaceutical, MSD and Sanofi.

Hiroyuki Nishiyama reports support for the present manuscript from Ono Pharmaceutical and Bristol-Myers Squibb; grants from Astellas Pharma, Ono Pharmaceutical, Takeda Pharmaceutical and Bayer and payment or honoraria from MSD, Chugai and Astellas Pharma.

Matthew D. Galsky reports support for the present manuscript from Ono Pharmaceutical and Bristol-Myers Squibb and grants to the institution from Janssen Oncology, Dendreon, Novartis, Bristol-Myers Squibb, Merck, AstraZeneca and Genentech/Roche; and consulting fees from BioMotiv, Janssen Pharmaceuticals, Dendreon, Merck, GlaxoSmithKline, Eli Lilly and Company, Astellas Pharma, Genentech, Bristol-Myers Squibb, Novartis, Pfizer, EMD Serono, AstraZeneca, Seagen, Incyte Corporation, Aileron Therapeutics, Dracen Pharmaceutical, Inovio Pharmaceuticals, Numab AG, Dragonfly Therapeutics, Basilea Pharmaceutica AG, UroGen Pharma, Infinity Pharmaceuticals and Gilead Sciences.

Federico Nasroulah reports support for the present manuscript from Ono Pharmaceutical and Bristol-Myers Squibb and is an employee of and holds stock in Bristol-Myers Squibb.

Sandra Collette reports support for the present manuscript from Ono Pharmaceutical and Bristol-Myers Squibb and is an employee of and holds stock or stock options in Bristol-Myers Squibb.

Edward Broughton reports support for the present manuscript from Ono Pharmaceutical and Bristol-Myers Squibb; support for attending meetings from Bristol-Myers Squibb; and is an employee of and holds shares in Bristol-Myers Squibb.

Keziban Ünsal-Kaçmaz reports support for the present manuscript from Ono Pharmaceutical and Bristol-Myers Squibb and is an employee of and holds stock or stock options in Bristol-Myers Squibb.

Yukinori Kamisuki reports support for the present manuscript from Ono Pharmaceutical and Bristol-Myers Squibb and is an employee of Ono Pharmaceutical.

Dean F. Bajorin reports support for the present manuscript from Ono Pharmaceutical and Bristol-Myers Squibb; grants from Bristol-Myers Squibb, Merck and Co, Novartis and AstraZeneca; consulting fees from Bristol-Myers Squibb; payment or honoraria from Novartis; and participation on a data safety monitoring board or advisory board for Merck and Co.

## Authors’ contributions

The authors had full access to all the data in the study and take responsibility for the integrity of the data and the accuracy of the data analysis.

Study concept and design: Yoshihiko Tomita, Federico Nasroulah, Sandra Collette, Edward Broughton, Keziban Ünsal-Kaçmaz, Yukinori Kamisuki and Dean F. Bajorin.

Provision of study materials or patients: Yoshihiko Tomita, Ko Kobayashi, Go Kimura, Mototsugu Oya, Hirotsugu Uemura, Hiroyuki Nishiyama, Matthew D. Galsky and Dean F. Bajorin.

Collection and assembly of data: Federico Nasroulah, Sandra Collette, Edward Broughton, Keziban Ünsal-Kaçmaz and Yukinori Kamisuki.

Data analysis and interpretation: All authors.

Drafting of the manuscript: Yoshihiko Tomita.

Critical revision of the manuscript for important intellectual content: All authors.

Final approval of manuscript: All authors.

## Supplementary Material

220713Clear_ONO-4538-33_CM274JPSub_finalSupplement_hyac155

## Data Availability

Qualified researchers may request Ono Pharmaceutical Co., Ltd. to disclose individual patient-level data from clinical studies through the following website: https://www.clinicalstudydatarequest.com/. For more information on the policy of Ono Pharmaceutical Co., Ltd. for the Disclosure of Clinical Study Data, please visit https://www.ono.co.jp/eng/rd/policy.html.

## Notes

Prior publication: The overall results of CheckMate 274 were published as Bajorin DF, Witjes JA, Gschwend JE, et al. Adjuvant nivolumab versus placebo in muscle-invasive urothelial carcinoma. N Engl J Med 2021;384:2102–14. Results of this analysis were presented at the 109th Annual Meeting of the Japanese Urological Association, December 7–9, 2021 (Yokohama, Japan).
