# Supplementary material for: Adjuvant nivolumab versus placebo following radical surgery for high-risk muscle-invasive urothelial carcinoma: a subgroup analysis of Japanese patients enrolled in the phase 3 CheckMate 274 trial
Source: Jpn J Clin Oncol. 2022 Oct 26;53(1):16–25. doi: 10.1093/jjco/hyac155 (PMC9825712; doi:10.1093/jjco/hyac155)
Supplement: 220713Clear_ONO-4538-33_CM274JPSub_finalSupplement_hyac155 [file 220713clear_ono-4538-33_cm274jpsub_finalsupplement_hyac155.docx]

**SUPPLEMENTARY MATERIALS**

**Table S1.** Demographic and clinical characteristics at baseline for Japanese patients with tumor PD-L1 expression of 1% or more

|  | **Nivolumab** | **Placebo** |
| --- | --- | --- |
|  | **(*n* = 11)** | **(*n* = 8)** |
| Age |  |  |
| Mean ± SD, years | 70.7 ± 12.5 | 64.5 ± 10.4 |
| <65 yr — no. (%) | 4 (36.4) | 4 (50.0) |
| ≥65 yr — no. (%) | 7 (63.6) | 4 (50.0) |
| Sex — no. (%) |  |  |
| Male | 9 (81.8) | 6 (75.0) |
| Female | 2 (18.2) | 2 (25.0) |
| ECOG PS at start of treatment — no. (%) |  |  |
| 0 | 7 (63.6) | 8 (100.0) |
| 1 | 4 (36.4) | 0 |
| 2 | 0 | 0 |
| Tumor origin at initial diagnosis — no. (%) |  |  |
| Urinary bladder | 6 (54.5) | 2 (25.0) |
| Renal pelvis | 4 (36.4) | 5 (62.5) |
| Ureter | 1 (9.1) | 1 (12.5) |
| Time from initial diagnosis to randomization — no. (%) |  |  |
| <1 yr | 10 (90.9) | 7 (87.5) |
| ≥1 yr | 1 (9.1) | 1 (12.5) |
| Cisplatin-based neoadjuvant chemotherapy — no. (%) | 4 (36.4) | 4 (50.0) |
| Pathological tumor stage at resection — no. (%)* |  |  |
| pTX | 1 (9.1) | 0 |
| pT0 | 0 | 0 |
| pTis | 0 | 0 |
| pT1 | 0 | 0 |
| pT2 | 0 | 0 |
| pT3 | 7 (63.6) | 7 (87.5) |
| pT4A | 3 (27.3) | 1 (12.5) |
| Node stage and node density — no. (%) |  |  |
| N+ | 6 (54.5) | 2 (25.0) |

*The pathological tumor staging included patients with N+, N0, or NX.

SD, standard deviation; ECOG PS, Eastern Cooperative Oncology Group performance status; PD-L1, programmed death ligand 1

**Table S2**. Demographic and clinical characteristics at baseline for patients with tumor PD-L1 ≥1%

|  | **Nivolumab** | **Placebo** |
| --- | --- | --- |
|  | **(*n* = 140)** | **(*n* = 142)** |
| Age |  |  |
| Mean (range) — yr | 64.4 (34‒92) | 65.9 (45‒84) |
| <65 yr — no. (%) | 67 (47.9) | 61 (43.0) |
| ≥65 yr — no. (%) | 73 (52.1) | 81 (57.0) |
| Sex — no. (%) |  |  |
| Male | 101 (72.1) | 112 (78.9) |
| Female | 39 (27.9) | 30 (21.1) |
| Race — no. (%) |  |  |
| White | 104 (74.3) | 109 (76.8) |
| Asian | 33 (23.6) | 28 (19.7) |
| Black | 0 | 2 (1.4) |
| American Indian/Alaska Native | 1 (0.7) | 0 |
| Other | 2 (1.4) | 2 (1.4) |
| Not reported | 0 | 1 (0.7) |
| ECOG PS — no. (%) |  |  |
| 0 | 86 (61.4) | 85 (59.9) |
| 1 | 51 (36.4) | 53 (37.3) |
| 2 | 3 (2.1) | 4 (2.8) |
| Tumor origin at initial diagnosis — no. (%) |  |  |
| Urinary bladder | 113 (80.7) | 117 (82.4) |
| Renal pelvis | 19 (13.6) | 14 (9.9) |
| Ureter | 8 (5.7) | 11 (7.7) |
| Time from initial diagnosis to randomization — no. (%) |  |  |
| <1 yr | 132 (94.3) | 129 (90.8) |
| ≥1 yr | 8 (5.7) | 13 (9.2) |
| Prior neoadjuvant cisplatin — no. (%) | 57 (40.7) | 61 (43.0) |
| Pathological tumor stage and nodal status at resection — no. (%)* |  |  |
| pT2N– | 8 (5.7) | 11 (7.7) |
| pT3-4N– | 72 (51.4) | 65 (45.8) |
| pT0-4N1 | 29 (20.7) | 33 (23.2) |
| pT0-4N2,3 | 31 (22.1) | 33 (23.2) |
| Pathological tumor stage at resection — no. (%)† |  |  |
| pTX | 4 (2.9) | 0 |
| pT0 | 3 (2.1) | 3 (2.1) |
| pTis | 0 | 0 |
| pT1 | 4 (2.9) | 2 (1.4) |
| pT2 | 19 (13.6) | 26 (18.3) |
| pT3 | 87 (62.1) | 83 (58.5) |
| pT4A | 23 (16.4) | 27 (19.0) |
| Not reported | 0 | 1 (0.7) |
| Node status — no. (%) |  |  |
| N0/X with <10 nodes removed | 38 (27.1) | 38 (26.8) |
| N0 with ≥10 nodes removed | 42 (30.0) | 38 (26.8) |
| N1 | 29 (20.7) | 33 (23.2) |
| N2 | 28 (20.0) | 26 (18.3) |
| N3 | 3 (2.1) | 7 (4.9) |

*This was not a pre-specified subgroup. pT2N- patients were only eligible if they received neoadjuvant cisplatin-based chemotherapy. N- includes N0/X. T0 includes pTX, pT0, and pTis.

†The pathological tumor staging included patients with N+, N0, or NX.

ECOG PS denotes Eastern Cooperative Oncology Group performance status, PD-L1, programmed death ligand 1

Reprinted from Ref.20. Copyright © 2021 Massachusetts Medical Society. All rights reserved.
